# Effect of selection for behavior on the blood glucose level in foxes exposed to restriction stress

**DOI:** 10.18699/vjgb-26-89

**Published:** 2026-10

**Authors:** R.G. Gulevich, S.G. Shikhevich, D.V. Shepeleva, A.V. Kharlamova

**Affiliations:** Institute of Cytology and Genetics of the Siberian Branch of the Russian Academy of Sciences, Novosibirsk, Russia; Institute of Cytology and Genetics of the Siberian Branch of the Russian Academy of Sciences, Novosibirsk, Russia; Institute of Cytology and Genetics of the Siberian Branch of the Russian Academy of Sciences, Novosibirsk, Russia; Institute of Cytology and Genetics of the Siberian Branch of the Russian Academy of Sciences, Novosibirsk, Russia

**Keywords:** selection, behavior, fox, aggressiveness, stress, glucose, tolerance, cortisol, correlation, отбор, поведение, лисицы, агрессивность, стресс, глюкоза, глюкозотолерантность, кортизол, корреляция

## Abstract

Selection of silver foxes for attitude to humans influences the hypothalamus-pituitary-adrenal axis and sympathetic nervous system, which, in turn, are involved in the regulation of carbohydrate metabolism under stress. It has also been shown that heredity affectes carbohydrate metabolism and glucose tolerance. It remains to find out whether the selection for attitude to humans affected carbohydrate metabolism and blood glucose levels in silver foxes. We studied basal blood glucose levels in tame, aggressive, and unselected male and female silver foxes and glucose levels in females exposed to stress. Our results indicate that the basal blood glucose level in tame foxes is significantly lower than in unselected or aggressive ones regardless of sex. In unselected males, this parameter was lower than in aggressive animals. Aggressive foxes showed a sex difference in the basal blood glucose level: it was higher in males than in females. The glucose levels in aggressive females remained higher than in tame ones immediately after 10-min stress and two hours afterwards. Only aggressive females showed an excess blood glucose level over the basal one two hours after stress. The bulk group of foxes demonstrated a negative correlation between blood glucose and behavior score (response to humans) both at rest and after stress. A glucose tolerance test (GTT) was performed to compare the rates of glucose absorption from the bloodstream in silver fox females selected for behavior. Although selection for behavior did not affect glucose tolerance, judging from the area under the GTT curves, aggressive females differed from tame and unselected at the beginning of the test in longer persistence of elevated blood glucose. Thus, the selection of silver foxes for attitude to humans influences blood glucose levels at rest in both sexes and after stress in females.

## Abstract (ru)

Известно, что отбор серебристо-черных лисиц по отношению к человеку влияет на гипоталамо-гипофизарно-надпочечниковую (ГГН) и симпатическую нервную системы, которые, в свою очередь, участвуют в регуляции углеводного обмена при стрессе. Наряду с этим показано влияние генетических факторов на углеводный обмен и толерантность к глюкозе. Оставалось неизвестным, влияет ли отбор по отношению к человеку на уровень глюкозы в крови у серебристо-черных лисиц. В данной работе исследовали базальный уровень глюкозы в крови у ручных, агрессивных и неселекционированных самцов и самок серебристо-черных лисиц, а также уровень глюкозы после рестрикционного стресса у самок. Полученные результаты свидетельствуют о достоверно более низком базальном уровне глюкозы в крови у ручных лисиц по сравнению с агрессивными и неселекционированными животными, независимо от пола. Кроме того, у неселекционированных самцов этот показатель был ниже по сравнению с агрессивными животными. У агрессивных лисиц найдены половые различия по базальному уровню глюкозы в крови: у самцов этот показатель выше, чем у самок. В течение всего исследуемого периода после стресса (через 10 мин и 2 ч) уровень глюкозы в крови у агрессивных самок оставался повышенным по сравнению с ручными. Спустя 2 ч после стресса только у агрессивных самок отмечали более высокий уровень глюкозы по сравнению с исходным уровнем. В суммарной выборке самок лисиц, независимо от поведения до и после стресса, найдена отрицательная корреляция между уровнем глюкозы в крови и баллом поведения, которым оценивали реакцию на человека. Для сравнения скорости поглощения глюкозы из общего кровотока у самок серебристо-черных лисиц, селекционируемых по поведению, проводили глюкозотолерантный тест (ГТТ). В целом, судя по показателям площади под кривыми ГТТ, отбор по поведению не влиял на толерантность к глюкозе, вместе с тем у агрессивных самок, в отличие от ручных и неселекционированных, в начале теста отмечали более продолжительный период повышенного уровня глюкозы в крови. Таким образом, отбор по поведению влияет на уровень глюкозы в крови у серебристо-черных лисиц независимо от пола в состоянии покоя, а у самок – и после стресса.

## Introduction

Glucose is the main energy source for the brain. An increased blood glucose level, ensuring stable delivery of the nutrient to the brain, is an important prerequisite for survival at stress (Magomedova, Cummins, 2016). It is known that stress activates the sympathoadrenal and hypothalamic-pituitary-adrenal (HPA) axes. Their signals are involved in the development of stress-induced hyperglycemia (Stover et al., 2002; Barth et al., 2007; Magomedova, Cummins, 2015; Ross et al., 2015). In particular, catecholamines and glucocorticoids can enhance gluconeogenesis (glucose synthesis from nonsugar precursors), glycogenolysis (glycogen conversion to glucose) in the liver, and glucose release from the liver. They also inhibit glucose uptake by cells from the bloodstream.

Hereditary differences in stress-induced hyperglycemia were noted in inbred mouse lines (Harizi et al., 2007). Movement restriction, considered to be psychic stress, was responded by BALB/c mice with the maximum blood corticosterone level, whereas the increase in glucose level was lower than in DBA or C57Bl/6 mice. In contrast, restricted C57Bl/6 mice showed the minimum increase in corticosterone and maximum increase in glucose as compared to DBA and BALB/c.

Blood glucose increase was also observed in Large White pigs after aggressive clashes of two unfamiliar castrated males. In these cases, blood glucose linearly correlated with both aggressiveness and catecholamine (epinephrine and norepinephrine) levels, but the cortisol level did not correlate with aggressiveness (Fernandez et al., 1994).A study of molecular mechanisms underlying the variation in stress sensitivity and aggressiveness was conducted in pigs of a synthetic line (SYN) obtained from males of one breed and hybrid dams from six commercial parental lines. Associations of DNA markers from ten genes related to the HPA axis were analyzed, as well as several physiological parameters: plasma levels of cortisol, creatine kinase, glucose, and lactate; adrenal weight; and aggressiveness (Muráni et al., 2010). A considerable association between single-nucleotide polymorphism g.233C>T in the CRH gene for corticotropin-releasing hormone and the plasma glucose level was revealed.

Low expression of genes involved in glucose metabolism, including glucose transport, gluconeogenesis, and glycolysis, was found in domesticated male pigs (or adapted to captivity for many generations) as compared to wild animals captured early in life. All animals received equal diets (He et al., 2017).

Studies of dogs of various breeds differing in liability to diabetes mellitus point to an influence of hereditary factors on hyperglycemia caused by insulin deficiency (Catchpole et al., 2013). This pancreatic hormone participates in the uptake of glucose from the bloodstream by cells and thereby lowers the blood glucose level. In contrast, small and fancy dog breeds are inclined to hypoglycemia. Its causes are thought to be disturbances in gluconeogenesis and glycogenolysis in the liver and low levels of glucocorticoids, which enhance these processes (Fleisher et al., 2008; Idowu, Heading, 2018).

Experiments on silver foxes selected for aggressive and tolerant attitude to humans may contribute to the understanding of the functional regulation of carbohydrate metabolism in animals with hereditary differences in the stress response of the HPA axis. The experiment modeling domestication, which has been conducted at the Institute of Cytology and Genetics (Novosibirsk) for decades, shows that silver fox selection for tame behavior lowers the HPA axis function as compared to animals selected for aggressive behavior (Bazhan et al., 1974; Oskina, 1996, 2008; Gulevich et al., 2004; Alexandrovich et al., 2023). In addition, the tame foxes show a lower activity of the sympathetic nervous system, as follows from the lower blood norepinephrine level in comparison to aggressive and unselected animals (Oskina et al., 2008).

The results obtained from Norway rats and American minks selected for tame and aggressive behavior indicate that selection for behavior may alter some carbohydrate metabolism parameters (Albert et al., 2008; Kalinina et al., 2022). Tame male rats demonstrated a decrease in blood glucose level in comparison to aggressive ones (Albert et al. 2008). There is no information on blood glucose in minks selected for behavior, but it has been shown that the activity of alpha amylase in the pancreas, duodenum, and jejunum in tame minks is higher than in aggressive ones. This enzyme degrades starch to di- and trisaccharides in the gastrointestinal tract, and these oligosaccharides are then degraded to glucose, which is absorbed into the bloodstream (Kalinina et al., 2022). The blood glucose level is influenced not only by the pancreatic alpha amylase activity but also by other multidirectional factors.

In addition, the maximum alpha amylase activity in the pancreas was recorded in minks with highest tame behavior scores and the minimum, in most aggressive animals. This correlation appears to be related to the fact that changes in the mink brain caused by selection for behavior, in particular, monoamine levels in the hippocampus (Kulikov et al., 2016), may correspond to behavior scores, and the signals induced by these changes may reach norepinephrine-secreting cells of adrenal medulla via the sympathicoadrenal axis and glucocorticoid-secreting adrenal cortex via the HPA axis. Both norepinephrine and glucocorticoids are involved in the secretory function of the pancreas (Mössner et al., 1989; Nagain-Domaine et al., 1999).

These studies leave open the question whether and how the selection of silver foxes for behavior affects blood glucose levels at rest and after stress.

The goal of the present study is to investigate the effect of selection for behavior on blood glucose levels in tame and aggressive silver foxes at rest and after stress.

## Materials and methods

Experiments were conducted with lines of adult silver foxes that had been selected for emotionally positive and aggressive-defensive attitudes to humans for over 60 years, hereafter referred to as tame and aggressive, respectively (Belyaev, 1979; Trut et al., 2004, 2009). A third group included foxes not exposed to deliberate selection for behavior, hereafter unselected.

Experimental animals were kept at the Shared Access Center for Gene Pools of Fur and Farm Animals (ICG, Novosibirsk). Animal behavior was assessed in the glove test. Tame foxes scored +4 to +4.5; aggressive, –2.5 to –3.5; and unselected, –0.75 to +1.Basal blood glucose levels were measured in mmol/L with a One Touch Basic Plus glucose meter in fox males and females of each group after overnight fasting. One blood drop from the ear tip was loaded onto the test strip. Each group included 12 males and 10 females.

The effects of 10-min restriction stress on blood cortisol and glucose levels were analyzed only in females, whereas males were used in other experiments. Each group included eight naïve females. In the restriction stress experiment, foxes were placed to animal carriers with one side made of wire mesh. To reduce the stress, the restriction was applied for shorter time (10 min) in comparison to earlier studies (20 min) (Oskina, 1996).

Blood samples for cortisol assay were taken from vena safena in the morning after overnight fasting. The sampling lasted no more than 2–3 min per animal. Blood was sampled immediately after 10-min stress and 2 h afterwards. After taking the first blood sample, the foxes were placed to their home cages. One week before the experimental stress, blood samples were taken for the basal cortisol level. Blood cortisol was assayed by enzyme immunoassay with a Cortisol-IFA-BEST kit (Vector-Best, Novosibirsk).

One month after blood sampling from vena safena and one week before the stress experiment, basal blood glucose was assayed in the same animals with a Satellite glucose meter (ELTA, Moscow) in the morning after overnight fasting. Anothert glucose measurement was done in females immediately after exposure to stress, and the animals were placed to their hame cages. The next measurement was done two hours after stress.

To analyze the rates of glucose uptake from the bloodstream after carbohydrate loading, the glucose tolerance test (GTT) was conducted in the fox groups: five naïve females from each group. Blood glucose levels were recorded after overnight fasting and 10, 20, 40, and 120 min after intravenous administration of 50 % glucose monohydrate (0.5 g/kg).

The statistical evaluation of the results was performed with Statistica 10.0 software (Stat Soft, United States). Data on the basal blood glucose levels in fox males and females from the tame, aggressive, and unselected groups were analyzed by ANOVA for independent measurements followed by Fisher’s LSD post-hoc test. Data on blood glucose levels in foxes within a particular behavior group in GTT and after stress were analyzed by Student’s dependent t-test, and foxes from different groups were compared by Student’s independent t-test.

## Results

Figure 1 presents data on the basal glucose level in peripheral blood of tame, aggressive, and unselected foxes of both sexes. Two-way ANOVA shows that selection for behavior influenced the blood glucose level (F2.60 = 16.07, p < 0.001), the sex factor did not (F1.60 = 1.64, p > 0.05), and their interaction was significant (F2.60 = 5.85, p < 0.01). The subsequent post-hoc test indicates that glucose levels in tame males were significantly lower than in unselected ( p < 0.05) or aggressive ( p < 0.001), and the levels in unselected males were lower than in aggressive ( p < 0.001). In tame females, as in males, glucose levels were lower than in unselected or aggressive ( p <0.05 in both cases). Sexrelated differences in glucose levels were recorded only in the aggressive group: males showed higher levels than females ( p < 0.001).

**Fig. 1.**
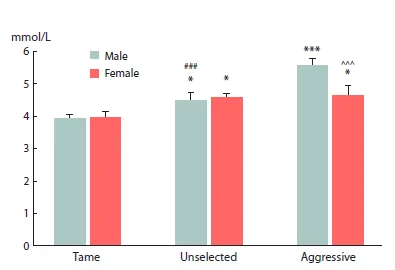
Basal blood glucose levels in tame, aggressive, and unselected male and female foxes, mmol/L. * p < 0.5, *** p < 0.001 compared to tame; ### p < 0.001 compared to aggressive; ^^^ p < 0.001 compared to corresponding males according to two-way ANOVA followed by post-hoc Fisher’s LSD test.

Figure 2 presents blood cortisol levels in tame, aggressive, and unselected fox females after restriction stress. This parameter was significantly lower in tame foxes than in aggressive or unselected before stress, immediately after stress, and two hours later. In tame foxes, 10-min restriction stress did not affect blood cortisol levels either immediately after stress or two hours after. In unselected and aggressive foxes, stress induced a significant increase in blood cortisol as compared to the respective basal levels ( p < 0.01, p <0.001, respectively). Two hours after stress, blood cortisol levels were significantly lower than those immediately after stress in unselected ( p < 0.01) or aggressive ( p < 0.001) foxes.

**Fig. 2.**
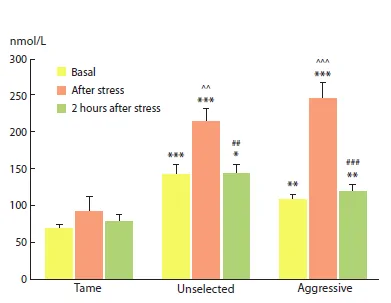
Blood cortisol levels (nmol/L) in tame, unselected, and aggressive female foxes after stress. * p < 0.05, ** p < 0.01, *** p < 0.001 as compared to tame females according to independent Student’s t-test; ^^ p < 0.01, ^^^ p < 0.001 as compared to the corresponding basal level according to dependent Student’s t-test; ## p < 0.01, ### p < 0.001 as compared to the level immediately after stress according to dependent Student’s t-test.

Figure 3 illustrates blood glucose levels in tame, aggressive, and unselected female foxes after restriction stress. We see that blood glucose levels in tame females were significantly lower than in aggressive before stress ( p < 0.01), immediately after stress ( p < 0.05), or two hours later ( p < 0.001). Glucose levels in unselected foxes were intermediate between tame and aggressive at all timepoints. The basal glucose level in unselected foxes did not differ from the corresponding levels in tame or aggressive ones, but after stress it was higher than in tame animals ( p < 0.01) and two hours after stress it was lower than in aggressive ones ( p < 0.001). Stress did not induce blood glucose changes in tame or unselected foxes throughout the experiment period. In aggressive animals, it increased as compared to the basal level two hours after stress ( p < 0.01).

**Fig. 3.**
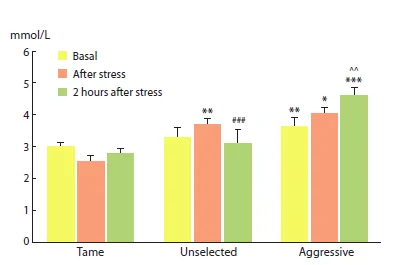
Blood glucose levels in tame, aggressive, and unselected female foxes after stress, mmol/L. * p < 0.05, ** p < 0.01, *** p <0.001 as compared to tame according to independent Student’s t-test; ^^ p <0.01 as compared to the corresponding basal level according to dependent Student’s t-test; ### p < 0.001 as compared to aggressive according to independent Student’s t-test.

The Table presents correlation coefficients between behavior scores and blood glucose levels in the overall group of foxes: tame, aggressive, and unselected. Significant negative correlations were found between blood glucose levels and behavior scores before stress, immediately after stress, and two hours after stress.

**Table 1.**
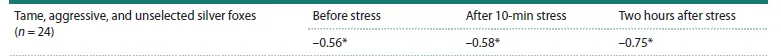
Correlation coefficients between behavior scores and blood glucose levels before and after stress in all foxes examined regardless of behavior Note. Behavior scores in unselected animals are indicated with plus and minus signs; tame, with plus signs; and aggressive, with minus signs. * Significance level for correlation coefficients p< 0.05.

We also assessed the correlation between blood glucose and cortisol levels in the same bulk sample. A significant positive correlation (0.51; n = 22) was found only at one timepoint: immediately after stress.

Data on blood glucose levels in tame, aggressive, and unselected female foxes in GTT are shown in Figure 4. Ten minutes after glucose monohydrate administration, blood glucose increased significantly in all animals regardless of behavior group: p <0.001 in tame and unselected, p <0.01 in aggressive.

**Fig. 4.**
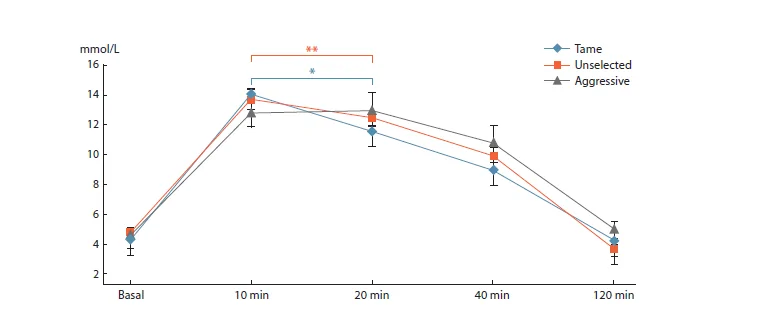
Blood glucose levels (mmol/L) in tame, unselected, and aggressive female foxes after administration of 50 % glucose monohydrate, 0.5 g/kg (glucose tolerance test). * p <0.05 and ** p < 0.01 – Student’s t-test for repetitive measurements for comparison of the levels between the 10- and 20-min timepoints.

Twenty minutes after test, glucose in tame and unselected foxes was significantly lower than the earlier values ( p <0.05 and p <0.01, respectively), but did not decrease in aggressive foxes, remaining elevated; thus, the elevated glucose after glucose monohydrate administration to aggressive foxes persisted longer than in tame or unselected ones. Only 40 min after the glucose monohydrate challenge, the glucose level in aggressive foxes became significantly lower than in the preceding measurements ( p < 0.05), whereas in tame and aggressive animals the decrease in comparison to preceding values continued (p <0.01 and p <0.05, respectively). Two hours after glucose monohydrate, blood glucose got back to a value close to the basal level in tame and aggressive foxes. In the unselected group, blood glucose at the same timepoint was significantly below the respective basal level ( p < 0.01) and lower than in the tame or aggressive groups ( p <0.01 and p <0.05, respectively).

The areas below the GTT curves did not differ significantly among the tame, unselected, and aggressive groups: 951.5 ± 77.7; 986.8 ± 83.3, and 1,088.1 ± 236.3, respectively. In contrast to the above results, GTT did not reveal significant differences in basal blood glucose levels in tame, aggressive, and unselected female foxes; apparently, because of smaller samples (n = 5).

## Discussion

Our results demonstrate a lower basal blood glucose level in tame male and female foxes as compared to unselected and aggressive ones (Fig. 1). This observation appears to

be caused by a lower activity of the HPA and sympathetic axes in the first group as compared to the other two, which has been reported by N.M. Bazhan et al. (1974), I.N. Oskina et al. (1985, 2008), and R.G. Gulevich et al. (2004). This study also confirms the lower basal cortisol level in tame females compared to aggressive or unselected ones (Fig. 2).

As mentioned above, glucocorticoids and norepinephrine participate in blood glucose regulation by intensifying gluconeogenesis (glucose synthesis from nonsugar precursors), glycogenolysis (glycogen breakdown to glucose) in the liver, and glucose release from the liver and also by inhibiting glucose uptake by cells from the bloodstream (Stover et al., 2002; Barth et al., 2007; Magomedova, Cummins, 2015; Ross et al., 2015). Studies on Norway male rats selected for behavior have shown that tame animals differed from aggressive in lower levels of blood corticosterone and glucose, which is consistent with data for foxes (Plyusnina, Oskina, 1997; Albert et al., 2008).

A lowered expression of genes involved in glucose me- tabolism, glucose transport, gluconeogenesis, and glycolylis has been found in domestic pigs as compared to wild ones captured early in life in spite of equal diets (He et al., 2017). Probably, fox selection for domestic and aggressive behavior influences the same glucose metabolism processes, so that blood glucose levels in tame foxes are lower than in aggressive ones.

The blood glucose levels measured in silver foxes in our study were close to those reported for dogs (3.3–6.2 mmol/L) by O. Idowu and K. Heading (2018). The gender difference in basal blood glucose levels found in aggressive foxes (higher values in males than in females) (Fig. 1) agrees with the results obtained on mice by H. Harizi et al. (2007), who presume that this sex dimorphism is due to the involvement of androgens in insulin resistance induction. Regrettably, the results on blood glucose in Norway rats selected for behavior include only males (Albert et al., 2008).

Restriction stress did not affect the cortisol or glucose levels in the blood of tame foxes, which showed a lower response to stress, at any timepoint: immediately after stress and two hours later. Blood cortisol levels in unselected and aggressive foxes increased significantly immediately after stress. Blood glucose levels did not change in unselected foxes but increased two hours after stress in aggressive ones, characterized by a stronger stress response.

In a study by P.D. Miles et al. (1991), i.c.v. carbachol (acetylcholine analog) injections to dogs raised the levels of catecholamines and cortisol, thereby mimicking the effect of stress, and enhanced not only glucose production but also its absorption. As a result, the glucose level increased slightly but statistically significantly no sooner than 15 min after the injection.

Thus, it is reasonable to suppose that the increase in blood glucose in stressed unselected and aggressive foxes occurred slightly later than after 10 min. The increase in blood glucose recorded in aggressive foxes two hours after stress as compared to the basal value could be caused by long-term intense production of glucose and/ or its release to blood combined with slower absorption from the bloodstream.

The negative correlation between blood glucose before and after stress, on the one side, and behavior score, on the other side, found in the bulk fox sample regardless of behavior (see the Table) is consistent with data on the correlation between blood glucose and aggressiveness in pigs after a clash between two unfamiliar males (Fernandez et al., 1994). The difference in the sign of correlation between foxes and pigs (negative in the former and positive in the latter) may be related to the fact that the behavior of the most aggressive foxes was marked with the minimum score and of the tamest, the maximum, as opposed to pigs. To put it simply, the higher is aggressiveness, the higher is the blood glucose level, both in foxes and pigs.

The same study on pigs demonstrated a correlation between the levels of glucose and catecholamines (epinephrine and norepinephrine) in blood. We have mentioned that the blood levels of norepinephrine in tame foxes is lower than in aggressive or unselected (Oskina et al., 2008).

The detected correlation between behavior scores and blood glucose levels in foxes is likely to be related to the assumption that the changes in the fox brain induced by selection for behavior, in particular, levels of tyrosine hydroxylase (enzyme involved in catecholamine production) and serotonin (Dygalo et al., 1988; Popova, 2017) correspond to behavior scores and the signals from such changes come to cells of the adrenal medulla, which secrete norepinephrine, via the sympathoadrenal axis and to the adrenal cortex, which secretes glucocorticoids, via the HPA axis. In turn, both norepinephrine and glucocorticoids participate in the regulation of gluconeogenesis in the liver, glucose release from the liver, and glucose uptake by cells from the bloodstream (Stover et al., 2002; Barth et al., 2007; Magomedova, Cummins, 2015; Ross et al., 2015).

The area under the GTT curve shows that the rate of glucose disposal after carbohydrate challenge did not differ among tame, aggressive, and unselected foxes. However, soon after glucose monohydrate administration to aggressive foxes, we noted a longer period of elevated blood glucose level than in tame or unselected animals, as it remained elevated after 20 min, whereas in tame and unselected foxes it decreased (Fig. 4).

The blood glucose level in tame and aggressive foxes returned to the basal level two hours after glucose monohydrate administration, whereas this parameter in unselected foxes remained significantly lower than the basal level and corresponding levels in tame or aggressive ones. This observation may be indicative of a faster glucose uptake from the bloodstream in unselected animals in this time interval. A slight hypoglycemia three hours after oral glucose administration has also been reported in dogs: the maximum values are recorded after 30–60 min, and the return to the basal level, 2 h after the challenge (Kaneko, 1997).

## Conclusion

The selection of silver foxes for attitude to humans affects the basal blood glucose level regardless of sex, which may be associated with changes in the activity of the sympathetic and HPA axes in the selection. Tame male and female foxes showed lower blood glucose levels than aggressive or unselected ones.

The stress conditions did not influence blood glucose in tame and unselected female foxes, whereas aggressive animals demonstrated a delayed response, as glucose levels rose only two hours after stress. Although the glucose tolerance estimated from the area under the GTT curve did not change in selection for behavior, a longer time interval of elevated blood glucose was noted at the early step of GTT in aggressive females than in tame or unselected.

Compliance with ethical standards. Experiments were conducted in accordance with the Guidelines on the work with laboratory predator mammals (GOST 33217-2014) and European bioethical standards (Directive 2010/63/EU).

## Conflict of interest

The authors declare no conflict of interest.
